# How Do Masters of Public Health Programs Teach Monitoring and Evaluation?

**DOI:** 10.3389/fpubh.2017.00136

**Published:** 2017-06-23

**Authors:** Himanshu Negandhi, Preeti Negandhi, Sanjay P. Zodpey, Hemali Kulatilaka, Radhika Dayal, Lauren J. Hart, Marybeth Grewe

**Affiliations:** ^1^Indian Institute of Public Health – Delhi, Public Health Foundation of India, Gurgaon, India; ^2^MEASURE Evaluation, University of North Carolina at Chapel Hill, Chapel Hill, NC, United States; ^3^Public Health Foundation of India, Delhi, India

**Keywords:** monitoring and evaluation, public health, competency, curriculum, masters program

## Abstract

**Introduction:**

The health systems in developing countries face challenges because of deficient monitoring and evaluation (M&E) capacity with respect to their knowledge, skills, and practices. Strengthening M&E training in public health education can help overcome the gaps in M&E capacity. There is a need to advance the teaching of M&E as a core element of public health education.

**Objectives:**

To review M&E teaching across Masters of Public Health programs and to identify core competencies for M&E teaching in South Asian context.

**Materials and methods:**

We undertook two activities to understand the M&E teaching across masters level programs: (1) desk review of M&E curriculum and teaching in masters programs globally and (2) review of M&E teaching across 10 institutions representing 4 South Asian countries. Subsequently, we used the findings of these two activities as inputs to identify core competencies for an M&E module through a consultative meeting with the 10 South Asian universities.

**Results:**

Masters programs are being offered globally in 321 universities of which 88 offered a Masters in Public Health, and M&E was taught in 95 universities. M&E was taught as a part of another module in 49 institutions. The most common duration of M&E teaching was 4–5 weeks. From the 70 institutes where information on electives was available, M&E was a core module/part of a core module at 42 universities and an elective at 28 universities. The consultative meeting identified 10 core competencies and draft learning objectives for M&E teaching in masters programs in South Asia.

**Conclusion:**

The desk review showed similarities in M&E course content but variations in course structure and delivery. The core competencies identified during the consultation included basic M&E concepts. The results of the review and the core competencies identified at the consultation are useful resources for institutions interested in refining/updating M&E curricula in their postgraduate degree programs. Our approach for curriculum development as well as the consensus building experience could also be adapted for use in other situations.

## Introduction

Monitoring and evaluation (M&E) provides information about the performance of government policies, programs, and projects. It can identify what works, what does not work, and provide information about why. M&E also provides information about the performance of governments, ministries and agencies, and managers and their staff (1). Evaluation is critical to public health programs locally and globally, as donors, governments, and others strive to improve program performance and validate their investments.^1^ M&E skills are particularly important for public health professionals. However, health systems in developing countries are challenged with deficient M&E capacity with respect to their knowledge, skills, and practices (2). In order to address this deficient capacity, there is a need to develop the supply of these skills to match demand as it grows among these countries (3). Public health trainings, especially among masters level programs, produce a large number of public health professionals across the world. Public health graduates are expected to assume several roles, including undertaking M&E. Strengthening M&E training in public health programs can help overcome the gaps in M&E capacity.

With public health education undergoing major reforms, the advent of the twenty first century has seen academic education move away from the traditional knowledge-based approach and the use of competency-based models is increasingly gaining relevance, since competency-based education has the potential to align the public health education program with health systems priorities (4, 5). As a response to national and global public health systems priorities, many public health institutes are developing and adopting new curricula to include contemporary solutions to public health issues. This shift toward competency-based public health education gained worldwide momentum after the publication of the seminal report on “Health Professionals for a new century: transforming education to strengthen health systems in an interdependent world” in 2010 (6). This report highlighted the need to undertake institutional and instructional reforms for responding to the challenges of the twenty first century. As a part of the instructional reforms, the report suggests adopting a competency-based curriculum to address public health challenges in diverse contexts (6). Competency-based education allows for a highly individualized learning process rather than the traditional, one-size-fits-all curriculum (7).

While competency frameworks can guide institutes to equip their graduates with the necessary skills to perform as effective public health professionals, there is a need to identify the competencies that underpin these functions. Many public and private sector institutions/universities across the globe offer masters level programs in public health that encompass the core elements of public health. Many schools of public health in India as well as in neighboring countries of Bangladesh, Nepal, and Sri Lanka offer Masters in Public Health (MPH) degree programs. Public health schools in these countries have engaged in collaborative efforts in public health education and public health research activities. However, our exploratory analysis of the curriculum of 34 Indian institutes offering MPH degrees showed that M&E competencies are often left out of masters level public health training. Efforts by individual schools to include M&E competencies were sporadic and not standardized.

A discussion on understanding competencies and curriculum-related issues for M&E is timely in the Asian context as many countries are moving toward revamping public health education. In this context, we undertook the present activity to review M&E teaching across Masters of Public Health programs and to identify core competencies necessary for M&E teaching in South Asian institutes.

## Materials and Methods

In 2013, we undertook two activities to understand the M&E teaching across masters level programs: (1) desk review of M&E curriculum and teaching in Masters programs globally and (2) review of M&E teaching across 10 institutions representing four South Asian countries. Subsequently, we used the findings of these 2 activities as inputs to identify core competencies for an M&E module through a consultative meeting with the 10 South Asian institutions.

### Desk Review of M&E Curriculum and Teaching in Masters Programs

We first compiled a country list using the online World Atlas^2^ and Wikipedia.^3^ We identified universities offering MPH programs in each country by using the Google search engine, SOPHAS portal,^4^ webometrics,^5^ Wikipedia,^6^ PubMed,^7^ Association of Schools of Public Health website,^8^ Google and Google Scholar,^9^ Cochrane library,^10^ and University libraries. The online search was restricted to English language. Key words for the search included “MPH, public health courses, department of health sciences, public health specialization, Masters of Public Health, Master in Public Health, public health universities, public health schools, MSc in Public Health.” We also contacted public health practitioners and personal contacts in the field of public health for information on any other institute offering M&E courses. The contact persons from individual institutes offering Masters programs were asked about other institutes offering similar programs in their countries. The process was repeated until no new institutes could be identified.

To obtain information about M&E teaching, we additionally searched university websites using key words such as “MPH/MSc syllabus/programs/curriculum, department of health sciences, Post Graduate MPH courses, monitoring, evaluation, M&E, M&E track, management, planning, implementation, program design in public health, university handbook, reports.” We included M&E teaching in any form, either as a separate module, as part of another module, or as a separate track within the Masters program. We obtained detailed information of M&E curricula within the Masters programs from university websites and brochures. All the data obtained were entered systematically into a matrix on an excel spreadsheet. The quantitative data were analyzed in the form of proportions.

Colleagues from MEASURE Evaluation (a project based at University of North Carolina, Chapel Hill, United States of America) independently undertook a review of postgraduate level M&E course content offered globally. This supplementary review was undertaken to provide necessary background information for PHFI to use in preparation for the consultative meeting.

### Review of M&E Teaching across 10 Institutions across Four South Asian Countries

We enlisted South Asian organizations/institutes offering public health programs identified through the desk review. Eventually, 10 institutes across India, Sri Lanka, Nepal, and Bangladesh were included in this review based on their willingness to participate. A questionnaire was mailed to each of these institutes to obtain additional information about the M&E teaching within their MPH programs (including pedagogy, topics covered under the M&E module, and the competencies acquired by the graduates at the end of the M&E course). All the information collected in the questionnaire was entered into an excel spreadsheet. The data were analyzed descriptively, mainly in the form of proportions.

### Consultative Meeting to Identify Core Competencies for an M&E Module

Two representatives from each of the 10 identified institutions from South Asia participated in a 2-day consultative meeting to identify core competencies for an M&E module. These participating individuals comprised public health experts, most of whom were in a senior teaching position within their institutions and had an expertise in monitoring and/or evaluation. The findings from the desk review and review of the M&E teachings across the 10 institutions from the four South Asian countries were deliberated upon among the participants. Additionally, the group also discussed the draft internal document on competencies for basic M&E training developed by Global Evaluation & Monitoring Network for Health (GEMNet-Health network has a mission of empowering member institutions to ensure access to quality M&E training, research, and services) during the discussions. We divided the participants into four groups while ensuring that no two participants from the same institute were in the same group. The objective of this group activity was to list core competencies and additional competencies that were important for MPH graduates. Each of the four groups listed the M&E competencies that they felt were “core” and “additional” to create a combined list of expected competencies. A voting exercise was undertaken to help individual participants choose M&E competencies that they felt were core (must include), additional (maybe included), and not to be included. At the end of the voting exercise, we listed the competencies in a matrix and discussed the statements. We removed duplicate statements and adopted a standard terminology across all statements until the group arrived at a consensus on the competency statements.

The desk review included online search for readily available curricula of different programs across the world, and a consultation for deliberations. This activity was carried out within commonly accepted educational settings, involving normal educational practices and instructional strategies. It did not involve any vulnerable groups, hence was exempted from ethics clearance.

## Results

### Desk Review of M&E Curriculum and Teaching in Masters Programs

Across 194 countries, 321 universities offering Masters programs were identified (Asia = 124, Europe = 57, North America = 68, South America = 12, Africa = 37, Australia = 23). Of these 321 universities, 88 offered an MPH, and M&E was taught in 95 universities (Asia = 23, Europe = 9, North America = 37, Africa = 13, Australia = 13). Some of these institutions offered masters level programs such as MSc Public Health, Masters in Monitoring and Program Evaluation, and Masters of Health Evaluation, which covered M&E components in their curriculum. Overall, these institutes teach M&E either as an individual track within the Masters program, as an independent module or as part of another module.

M&E was taught as part of another module across 49 institutions. These modules ranged from health systems management, planning and financing, economic evaluation, health informatics, health promotion and behavioral sciences, health care systems and policy development to population health, public health leadership, and management and research methodology. Ten institutions covered it as an independent track within the Masters program, while 41 programs covered it as an independent module. The most common duration of M&E teaching (where available) was 4–5 weeks. From the 70 institutes where information on electives was available, M&E was a core module/part of a core module at 42 universities and an elective at 28 universities.

Of the courses for which information was available, 57.9% were on-campus classroom-based programs; few distance-learning/online teaching and learning courses for M&E were also offered (4.2%). Some universities (9.5%) offer a combination of both, on-campus and off-campus M&E teaching. The pedagogic methods to teach M&E were multimodal and included lectures, case studies, tutorials, seminars, group work, individual assignments, e-learning, workshops, and audio–visual clips. Common student evaluation methods included a theory examination, problem-solving assignment (individual/group), dissertation/project/thesis, developing a program evaluation plan, evaluation of a national health program, etc. There was overlap between the listed competencies and the topics covered in the syllabus. A list of the most common competencies across different institutions are as follows:

- Define the planning cycle with a specific focus on M&E of programs.
- Determine the need for conducting program evaluation and develop goals.
- Define and describe the steps in planning a health program, including M&E.
- Develop skills of planning and management through M&E of public health programs.
- Conceptualize and design an evaluation program and conduct the evaluation, including pretesting and data collection, data analysis, and interpretation and application of the results relevant to their area of work/interest in a real-world setting.
- Make appropriate evaluation choices suited to a range of scenarios.
- Demonstrate the use of M&E tools and techniques for planning.
- Gain experience in/understand and apply major M&E frameworks, models, and approaches for evaluation of public health programs in different contexts.
- Critically appraise a range of evaluations.
- Write real-world health and population-level evaluation proposals.

### M&E Teaching across 10 Institutions from 4 South Asian Countries

The 10 institutions from South Asia that participated in the consultation included (i) BRAC University, Dhaka; (ii) National Institute of Preventive and Social Medicine (Bangladesh), (iii) Datta Meghe Institute of Medical Sciences, Sawangi, Wardha; (iv) Manipal University, Manipal; (v) National Institute of Epidemiology, Chennai; (vi) Public Health Foundation of India, New Delhi; (vii) Tata Institute of Social Sciences, Mumbai (India), (viii) BP Koirala Institute of Medical Sciences, Dharan; (ix) Institute of Medicine, Kathmandu (Nepal), and (x) University of Kelaniya, Colombo (Sri Lanka).

The duration of the MPH program was 2 years in 6 out of the 10 institutes. Of the remaining four institutes, two offered a 1-year MPH, while two other institutes offered a 1.5-year MPH. All institutes were either accredited or affiliated with a university for their MPH programs. Seven institutes covered M&E teaching as part of some other module such as health system management, research methodology, health management information systems, epidemiology, or public health planning. At BP Koirala Institute of Medical Sciences, Nepal, M&E was included as an elective internship for 3 months and included a posting in the district health system for Public Health Practice. M&E was an independent module at PHFI and Manipal University, while BRAC University offered M&E as an independent module as well as a separate track within the MPH program.

**Figure 1:**
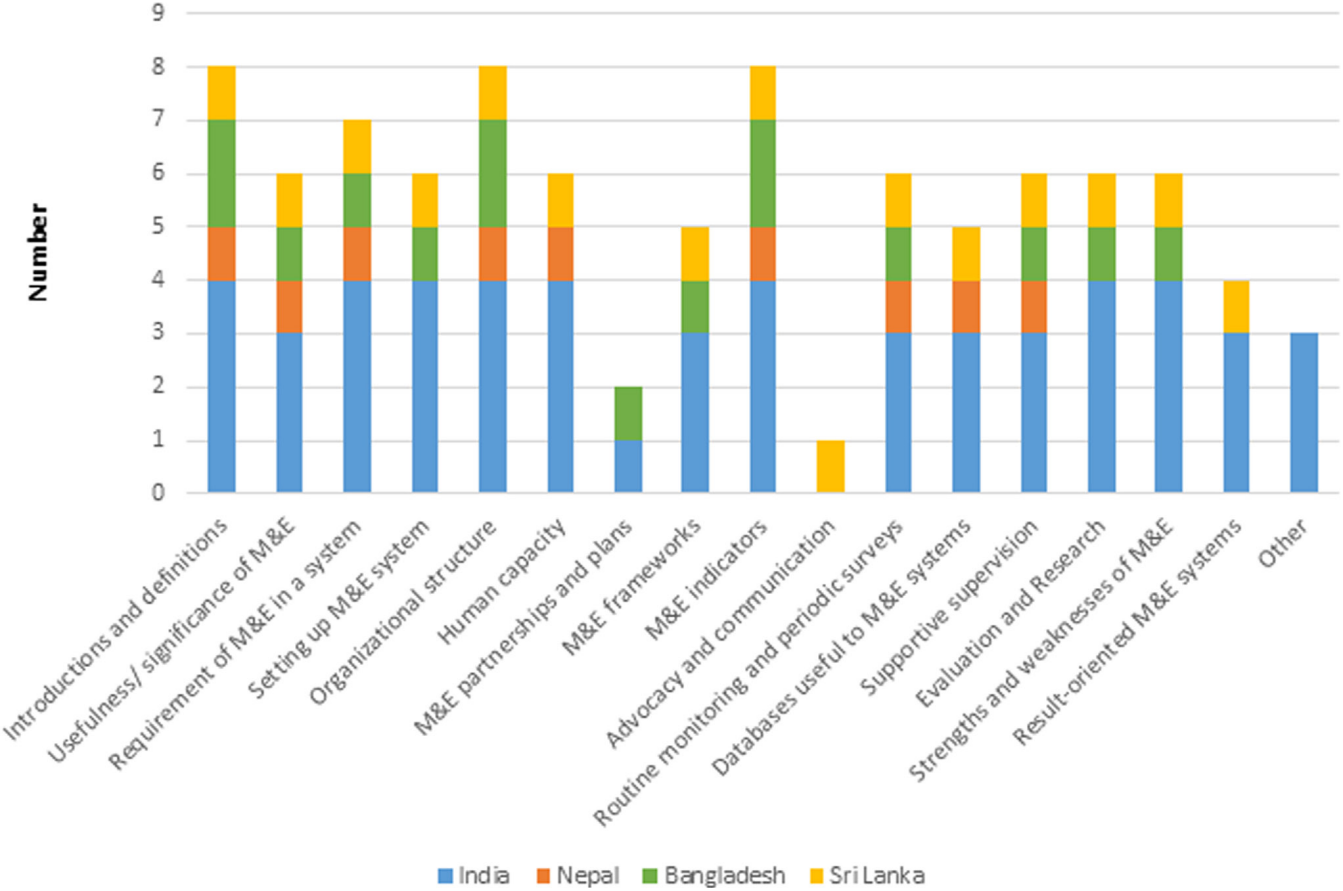
Monitoring and evaluation (M&E) topics covered by select institutes of four South Asian countries.

The duration of M&E instruction ranged from as short as 5 days to an entire semester of 6 months. At a few institutes, almost two-thirds of the entire duration of M&E teaching was dedicated to field practicum. It was evident from the information provided by the institutes that field work/project/dissertation was an important component of instruction as it drew on students’ M&E skills and was included in the curriculum at most institutes. The data on topics covered in M&E teaching were available for eight institutes (PHFI was excluded because the program was not launched at the time of the review) and are presented country-wise in Figure 1.

The pedagogic methods applied to M&E teaching across these institutes were varied. These included didactic lectures, assignments, field practicum/field visits, group discussions, case studies, journal clubs, mock evaluations, interactive workshops, presentations, and internship. Most institutes also evaluated students based on a project that drew on their M&E skills. At program completion, students were commonly expected to:

- Demonstrate an understanding of, and apply the principles of needs assessment and analysis in public health.
- Use available data sources and data analysis and synthesis techniques related to needs assessment functions.
- Articulate the use of needs assessments for program planning, implementation, evaluation, and modification.
- Explain assessment methodologies and techniques for both external (community) and internal (organizational) uses, use program planning principles, and strategies in public health.
- Apply the steps involved in translating needs assessment information into public health policy.
- Conceptualize the elements of health systems to effectively design, develop, implement, and evaluate public health interventions.

A 2-day consultation was held in September 2013 at New Delhi with representation from the 10 identified South Asian institutions. In order to identify core/essential competencies for an M&E module, the group reviewed the findings from the PHFI and MEASURE Evaluation global reviews of M&E curriculum; review of M&E teaching across the 10 South Asian institutions; and the draft GEMNet-Health competency document. The group identified the following 10 competencies as core competencies:

- Ability to comprehend basic M&E concepts and the importance of M&E and differentiate between M&E.
- Ability to identify and design M&E frameworks.
- Ability to collect, manage, analyze, and interpret data.
- Ability to identify and develop indicators.
- Ability to develop and use M&E tools.
- Ability to identify and engage stakeholders at all levels.
- Ability to assess data quality.
- Ability to use M&E data in decision-making.
- Ability to identify appropriate evaluation design and method.
- Ability to write reports, communicate, and disseminate M&E information.

After reaching upon an agreement on the 10 core competencies for the M&E curriculum, the group also developed a draft list of learning objectives for these 10 competencies and a short-term plan for their own institute to incorporate these competencies in their syllabi.

## Discussion

There were many similarities between the different M&E courses included in this review. First, there was a great deal of similarity between M&E course content at different global universities. Such similarity between M&E course content globally can be expected largely on the basis of perspective and knowledge base that M&E courses are expected to cover. A majority of programs were carried out over the course of 4–5 months or one semester. This is expected as modular teaching across most global universities that follow a semester-based pattern where students take multiple modules to complete the credit requirements for a semester. A large number of institutes included in the study offered M&E teaching on-campus; online/distance education courses were very few. This was surprising given the wide reach and applications of distance-learning modules in MPH curriculum globally. While anecdotal information suggests that core modules with higher credits (like epidemiology, biostatistics, and public health principles) are offered in a dual-mode (virtual and in-person) across many MPH programs, M&E teaching is still more conventional in its offering. This could stem from the higher complexity and application-based content of M&E modules that makes it challenging to design and deliver a fully online module.

There was large variability in the course structure of M&E teaching in our review. Courses differed in several ways; there were inclusions of evaluation of ethical, cultural and political issues in public health in some courses, albeit context- and country-specific. This is important from the context of M&E teaching as the students need to contextualize the M&E methods and apply it within their unique health system context. While many courses included working in groups or on projects, only one-third of courses included practical experience as a competency. As such, even though country and context-specific competencies are not included in many curricula, students may indeed be acquiring related skills through their group work or project work. Most courses did not focus on country-specific issues; it would be interesting to further explore how M&E coursework is or could be better tailored to different student populations or practical needs. The use of tools and resources was largely unaddressed as a competency or a topic. This could translate into MPH graduates possessing limited knowledge about preexisting M&E tools and resources. There were wide variations in the emphasis on different topics across the curriculum. For example, course time devoted to data analysis varied (some covered data analysis generally; some spent a significant portion of the course covering statistical issues or methodologies).

The very design of a competency-driven curriculum for an M&E module in MPH programs remains a challenge. This is because M&E is an application-driven subject that draws upon the graduates’ knowledge base from a variety of other modules offered in the MPH program, ranging from modules on basic public health principles to health systems functioning and across the spectrum of qualitative to quantitative research methods. In the presence of such wide overlap, it is natural for individual Masters programs to offer the relevant M&E components across a group of modules rather than as a part of a specific module. Alternately, M&E can be offered as a capstone module in the last semester of the MPH program where it brings together all the relevant theory and prepares the graduates to apply their learnings across the health system. Institutes could also proceed in creating a separate M&E track that could provide a more systematic and detailed understanding of the subject from its basics to its application. Individual schools will have to undertake a multi-stakeholder analysis to understand the dynamics of teaching the M&E content is these different forms.

The overall focus of the curriculum content centered more on evaluation than on monitoring. Due to the more advanced methods used in evaluation, it may be that there are more often courses specifically dedicated to evaluation, while monitoring is usually included within a larger discussion of M&E, program planning and management, or program assessment. It is also likely that the search terms we used in the global review disproportionately picked up evaluation, rather than monitoring.

With particular reference to institutes offering MPH programs in South Asia, the participating institutions highlighted the need for sharing knowledge and experience across the institutions. This type of institutional and instructional support, through collaboration, can provide technical guidance for conducting workshops; financial support for meetings/curriculum-re-design; development of internship exchange programs; guidance for updating existing M&E modules; opportunities for faculty development; and assistance in online course development. The global M&E curriculum review and the identification of core M&E competencies are useful inputs for designing an M&E module that meets the needs of these South Asian countries. Successful acquisition of competencies from improved instruction will equip graduates with relevant skills to undertake M&E activities at their workplaces. This consultation provided a common platform for institutes across these four countries to share and connect for future endeavors.

This list of core competencies for M&E teaching in Masters programs evolved after a consultative dialog between academics and is informed by a comprehensive review of global M&E teaching across Masters programs. These could serve as a template that may be adopted by other countries, particularly from Asia or developing countries across the globe. Although the health system context and needs across countries vary, in our opinion, the overall competencies identified by these four countries could be applicable in other settings. Additionally, if countries plan to develop their own set of competencies, our methodology may guide their efforts.

Monitoring and evaluation is a core element of public health and its teaching should be included in public health education. This will enable public health professionals to better confront and resolve system-based and context-specific M&E challenges. The role of a strong M&E framework in designing programs, selecting an appropriate intervention, delivery of the intervention, and its M&E is backed by evidence from within the health systems. Maternal and Child Health Integrated Program introduced mHealth for integrating mobile technology into health programs and developed a global M&E framework for program implementers to help them develop national M&E plans to monitor the implementation of the program (8). M&E can and should be integrated into the daily work of health professionals and other relevant stakeholders. Once set up, these systems can generate data and information allowing for greater transparency and accountability and help identifying lessons learned. It lists an example about how an evaluation of a domestic violence intervention in the maternity and sexual health services in a UK hospital helped the partners built on the results of the evaluation to further improve the intervention.^11^ All public health interventions, which eventually get monitored and evaluated, operate within a sociocultural–political milieu and this should be included in M&E teaching. Their role becomes particularly relevant within the context of evaluation studies.

Several different factors limited the results of this work. We looked only at M&E teaching leading to a Masters degree. Some universities offered short courses or workshops that have been excluded from this review. This review is predominantly driven by a secondary search and did not seek the perspective of the students. The review draws upon information that is available in public domain, or was amenable to search by the study team. Some excluded institutes had limited information readily available on their websites, especially in developing countries. Their curricula could not be accessed without the use of a valid username and password. There were instances of some websites that were last updated more than 3 years ago. Although we used Google Translate, there were many non-English programs, especially in Latin America, where we did not translate the course details to English. The list of core competencies developed during the consultation predominantly reflected the views of developing countries, particularly from South Asia, as the opinion of the consultation participants reflected the health system context and needs in their individual countries.

## Conclusion

The desk review found that there were similarities in the M&E course contents, but variations in the course structure and delivery. During the consultation, 10 core M&E competencies were identified. These included core M&E concepts including indicators, tools for data collection, analyses, frameworks, etc.

The desk review results and the core M&E competencies identified at the consultation are useful resources for institutions interested in refining/updating M&E curricula in their postgraduate degree programs. Our approach for curriculum review as well as the consensus building experience for identifying core M&E competencies could also be adapted for use in other situations. M&E is being recognized as a core discipline of public health, and its role is evident in the context of M&E of public health interventions.

The partnerships built through this process could contribute to other collaborative activities for the consortium of South Asian Universities, such as sharing of knowledge, M&E capacity building for faculty, development of M&E course outlines for MPH programs; and identifying core competencies and topics for M&E tracks/concentrations in MPH programs.

## Author Contributions

HN, PN, SZ, HK, and RD were involved in the conceptualization, design and conduct of the review, as well as drafting the manuscript. LH and MG provided technical inputs to the manuscript and helped in finalizing the manuscript.

## Conflict of Interest Statement

The authors declare that the research was conducted in the absence of any commercial or financial relationships that could be construed as a potential conflict of interest.

## Footnotes

^1^*Global Health Systems and Development*. (2011). Available from: http://www2.tulane.edu/publichealth/ghsd/upload/GHSD_VIEWBOOK_112011.pdf.

^2^*Countries Listed by Continent*. (2014). Available from: http://www.worldatlas.com/cntycont.htm#.Uda9w_kzgbg.

^3^*Professional Degrees of Public Health*. (2014). Available from: http://en.wikipedia.org/wiki/Professional_degrees_of_public_health.

^4^Sophas.org. Available from: http://sophas.org/.

^5^*Ranking Web of Universities*. (2014). Available from: http://www.webometrics.info/en/.

^6^*Wikipedia*. (2014). Available from: https://en.wikipedia.org/wiki/Main_Page.

^7^PubMed.gov. US National Library of Medicine, National Institutes of Health. (2014). Available from: https://www.ncbi.nlm.nih.gov/pubmed/.

^8^Association of Schools and Programs of Public Health. (2014). Available from: http://www.aspph.org/program-finder/?degree=MPH.

^9^Google Scholar. (2014). Available from: https://scholar.google.co.in/.

^10^Cochrane Library. (2014). Available from: http://www.cochranelibrary.com/.

^11^*Strengthening Health System Responses to Gender-Based Violence in Eastern Europe and Central Asia: A Resource Package*. (2017). Available from: http://www.health-genderviolence.org/guidance-for-health-care-professionals-in-strengthening-health-system-responses-to-gender-based-v-15.
